# The role of glucocorticoids in increasing cardiovascular risk

**DOI:** 10.3389/fcvm.2023.1187100

**Published:** 2023-07-05

**Authors:** Hai-Wei Deng, Wei-Yi Mei, Qing Xu, Yuan-Sheng Zhai, Xiao-Xiong Lin, Jie Li, Teng-Fei Li, Qian Zheng, Jin-Sheng Chen, Shun Ou-Yang, Zhi-Bin Huang, Yun-Jiu Cheng

**Affiliations:** ^1^ Department of Cardiology, The First Affiliated Hospital, Sun Yat-Sen University, Guangzhou, China; ^2^ Key Laboratory on Assisted Circulation Ministry of Health, The First Affiliated Hospital, Sun Yat-Sen University, Guangzhou, China; ^3^ Department of Cardiology, Guangdong Cardiovascular Institute, Guangdong Provincial People’s Hospital, Guangdong Academy of Medical Sciences, Guangzhou, China; ^4^ The Second School of Clinical Medicine, Southern Medical University, Guangzhou, China

**Keywords:** glucocorticoids, cardiovascular risk, coronary heart disease, heart failure, stroke, all-cause death

## Abstract

**Introduction:**

Different studies provide conflicting evidence regarding the potential for glucocorticoids (GCs) to increase the risk of cardiovascular diseases. This study performed a systematic review and meta-analysis to determine the correlation between GCs and cardiovascular risk, including major adverse cardiovascular events (MACE), death from any cause, coronary heart disease (CHD), heart failure (HF), and stroke.

**Methods:**

We performed a comprehensive search in PubMed and Embase (from inception to June 1, 2022). Studies that reported relative risk (RR) estimates with 95% confidence intervals (CIs) for the associations of interest were included.

**Results:**

A total of 43 studies with 15,572,512 subjects were included. Patients taking GCs had a higher risk of MACE (RR = 1.27, 95% CI: 1.15–1.40), CHD (RR = 1.25, 95% CI: 1.11–1.41), and HF (RR = 1.92, 95% CI: 1.51–2.45). The MACE risk increased by 10% (95% CI: 6%–15%) for each additional gram of GCs cumulative dose or by 63% (95% CI: 46%–83%) for an additional 10 μg daily dose. The subgroup analysis suggested that not inhaled GCs and current GCs use were associated with increasing MACE risk. Similarly, GCs were linked to an increase in absolute MACE risk of 13.94 (95% CI: 10.29–17.58) cases per 1,000 person-years.

**Conclusions:**

Administration of GCs is possibly related with increased risk for MACE, CHD, and HF but not increased all-cause death or stroke. Furthermore, it seems that the risk of MACE increased with increasing cumulative or daily dose of GCs.

## Introduction

Since their discovery in the 1930s, glucocorticoids (GCs) are an essential class of anti-inflammatory and immunosuppressive drug widely used for treating medical conditions such as asthma and chronic obstructive pulmonary disease, rheumatoid arthritis, inflammatory bowel disease, and lymphoid malignancies (1–5). It has been well recognized that the use of GCs may lead to adverse events, such as cardiovascular diseases (CVDs) (6, 7). Although numerous studies support this idea, evidence of increased adverse cardiovascular events remains conflicting. Schultz and Innala reported an increased risk of CVDs associated with GCs intake (8, 9). However, other studies failed to explore a significant relationship between GCs and adverse cardiac events (10, 11). In contrast, GCs were observed to reduce cardiovascular and all-cause mortality in women (12). Differences in GCs type and dose, population characteristics, study design, outcome type, and the number of events may account for inconsistencies among studies.

With these inconsistent findings in mind, the cardiac safety of individual GCs required further investigation to guide clinical decisions better. Therefore, we performed a systematic review and meta-analysis of published studies to comprehensively determine the correlation between GCs and CVDs, including major adverse cardiovascular events (MACE), death from any cause, coronary heart disease (CHD), heart failure (HF), and stroke. We tested the hypothesis that an increased dose of GCs use is related to a greater risk of MACE.

## Methods

### Search strategy

For the meta-analysis, the Preferred Reporting Items for Systematic Reviews and Meta-Analyses (PRISMA) checklist was used (13). The trail was registered with PROSPERO identifier CRD42022330612. We performed a comprehensive search in PubMed and Embase (from inception to June 1, 2022) for all full-text articles using the following text and keywords in combination: corticosteroid, fluticasone, budesonide, mometasone, prednisone, prednisolone, methylprednisolone, hydrocortisone, betamethasone, glucocorticoid, triamcinolone, flunisolide, beclomethasone, ciclesonide, flunisolide, inhaled corticosteroids (ICS), cardiovascular disease, cardiovascular events, stroke, cerebrovascular accident, cerebrovascular disease, coronary heart disease, myocardial infarction, coronary artery disease, ischemic heart disease, ischaemic heart disease, acute coronary syndrome, and heart failure. The search was limited to humans, but no restrictions were applied based on language, gender, or location. References from included studies and correlated reviews were manually searched to identify additional relevant studies. Duplicate data were removed for studies reporting information from the same cohort for the same outcome. When duplicate studies revealed data from different outcomes, they were included in the pooled analysis.

### Inclusion and exclusion criteria

To minimize differences between studies, the inclusion criteria were as follows: (1) studies presented the use of GCs (users and non-users), including cohort, case-control studies, and randomized controlled trials; (2) studies reported outcomes of CVDs with the minimum information necessary to estimate the relative risk (RR). Moreover, we excluded review articles, letters to editors, and case reports. The decision for inclusion of each study was made independently by two authors. Conflicts between reviewers were resolved by consensus.

### Data extraction and quality assessment

Two authors independently extracted relevant data such as the year of publication, geographical location, source of study, study design, baseline patient characteristics, the definition of exposure, the outcome of CVDs, and the cumulative and daily dose of GCs use. We assessed the quality of observational studies using the Newcastlee-Ottawa scale (14). We assessed the quality of randomized controlled trials using the modified Jadad score (15), with a higher score indicating higher quality of the study.

The primary outcome of this study was MACE, including CHD, stroke, HF, and cardiovascular death. Additionally, we examined CHD, HF, and stroke all-cause death as secondary outcomes.

### Statistical analysis

The primary measure of the association between GCs and CVDs in each study was relative risks (RR). Because the incidence of CVDs is rare, hazard ratio (HR) and odds ratio (OR) were treated as equivalent estimates of RR (16–18). We extracted the adjusted estimates when both unadjusted and adjusted estimates were provided.

Random effects were used to calculate pooled RRs (95% CI) to account for heterogeneity among studies, and the results are treated as conservative estimates because CIs are wider. Cochran’s Q and I2 statistics were used to quantify heterogeneity across estimates, with an I2 greater than 50% indicating significant heterogeneity (19). Meta-regression and stratified analyses were used to assess the potential sources of between-study heterogeneity. Studies were stratified based on study design (case-control, cohort, or randomized controlled trial), events (<1,000 or >=1,000), type of GCs (inhaled or not inhaled), location (Asia, Europe, or North America), age (<65 or >=65 years), male proportion (<50% or >=50%), time periods of GCs use (ever or current), and whether risk profiles were adjusted. Moreover, the treatment of GCs changed in 2010. The European League Against Rheumatism (EULAR) recommended adding glucocorticoids to DMARD monotherapy for rheumatoid arthritis as the initial treatment (20). We therefore selected 2010 as the cut-off point for subgroup analysis of the year of publication. Funnel plot, Begg’s test, and Egger’s test were used to evaluate publication bias (21).

In the dose-response analysis, the generalized least squares (GLST) regression model and restricted cubic splines were used to assess pooled dose-response relation between GCs and MACE. Both linear models and nonlinear models were fitted, and the results presented 95% CIs (22, 23). While exposure was reported as categorical data with a range, the mean or median exposure was extracted for each category, and the midpoint was used. Similarly, when the lowest or highest categories were open-ended, the width of the category was assumed to be the same as the adjacent category when estimating the midpoint.

We calculated the absolute difference in risk of GCs treatment as [(RR −1)* I_0_]. RR indicates pooled RRs and I_0_ was the cumulative incidence of events among patients without GCs (24).

All statistical analyses were performed using STATA 15.0 (STATA Corporation, College Station, TX). A two-sided *p*-value <0.05 was considered statistically significant.

## Results

As displayed in Supplementary Figure S1, of 7,422 potentially relevant papers initially screened, 54 were considered of interest, and the full text was retrieved for detailed evaluation. Finally, 43 eligible studies that enrolled 15,572,512 subjects (ranging from 182 to 14,467,072 in each study) were included in the meta-analysis.

The studies included 33 population-based cohort studies, nine case-control studies, and one randomized controlled clinical trial. Among the included studies, five were from Asia, 16 from Europe, and 22 from North America. Data for cumulative and daily GCs doses were available in 7 and 11 studies, respectively. The main characteristics, including references to the studies, were presented in Supplementary Table S1.

As shown in Supplementary Table S2, based on the Newcastle-Ottawa quality assessment scale for cohort studies and case-control studies, they got at least 5 points, indicating an overall good quality. Similarly, the only randomized controlled trial was also high, with a modified Jadad score of 5.

### Association between GCs and MACE

**Figure 1:**
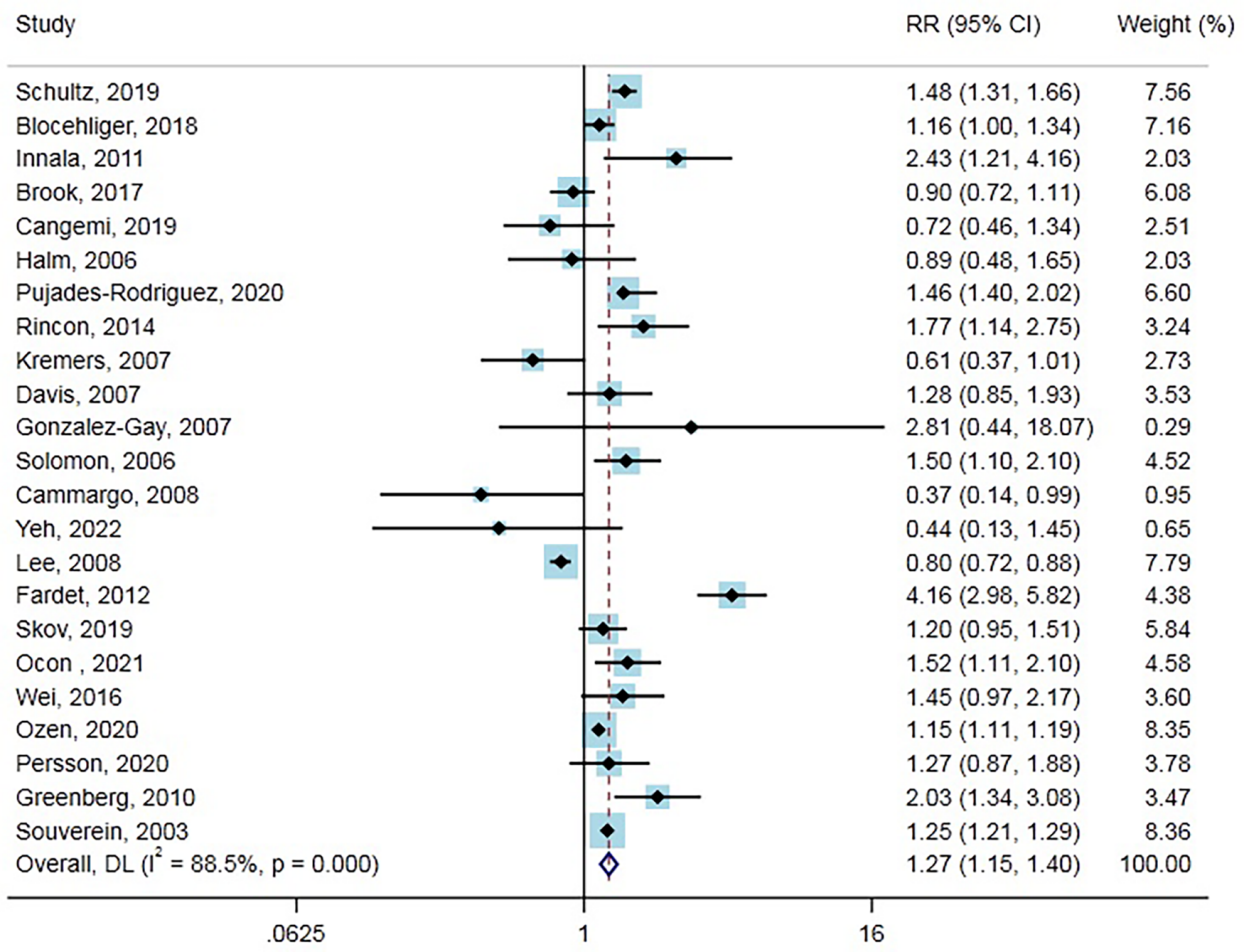
Forest plot showing the RR of MACE.

Twenty-three studies (17 cohort studies, five case-control studies, and one randomized controlled trial) with 883,877 cases reported the outcome of MACE. Overall, GCs use was significantly associated with increased MACE risk compared with no use (RR = 1.27, 95% CI: 1.15–1.40, Figure 1). Heterogeneity analyses suggested considerable heterogeneity across studies (Cochran’s *Q* = 190.64, *I*^2^ = 88.5%, *P* < 0.001). Therefore, subgroup analysis and meta-regression were performed in different subgroups across various main study characteristics and clinical factors to identify potential sources of heterogeneity (Table 1). The difference in the type of study, number of events, location, baseline disease, age, the time periods of GCs use (current or ever), and whether risk profiles were adjusted or publication year did not contribute to the heterogeneity. In contrast, the type of GCs and male proportion were associated with heterogeneity. Notably, increased risk of MACE associated with GCs was not observed in some of the subgroup analyses, including a subgroup of study type (case-control study, randomized controlled trial), the number of events (<1,000), location (Asia), baseline disease (chronic obstructive pulmonary disease/asthma, etc.), Age (>=65 years), Male proportion (>=50%), time periods of GCs use (ever), adjusted for risk profiles (no), publication year (<2010). In the subgroup analysis, we found that current GCs users had a significantly higher risk of developing MACE than non-GC users (RR = 1.37, 95% CI: 1.27–1.49), while the ever GCs users did not. Furthermore, the risk of MACE was lower in patients who used inhaled GCs (RR = 0.82, 95% CI: 0.69–0.97). Also, from nine cohort studies that reported on person-years in GCs users and non-GCs users, we calculated an absolute MACE risk increase of 13.94 (95% CI: 10.29–17.58) cases per 1,000 person-years in GCs users. Moreover, results from Funnel plots (Supplementary Figure S2A), Egger’s test (*P* = 0.686), and Begg test (*P* = 0.751) revealed no evidence of publication bias.

**Table 1:** Stratified analysis and Heterogeneity Analysis of RRs.

	MACE		All-cause Death		CHD		Heart Failure		Stroke	
Factors Stratified	RR (95% CI)	*p* Value	RR (95% CI)	*p* Value	RR (95% CI)	*p* Value	RR (95% CI)	*p* Value	RR (95% CI)	*p* Value
All studies	1.27 (1.15–1.40)		0.98 (0.76–1.25)		1.25 (1.11–1.41)		1.92 (1.51–2.45)		1.10 (0.95–1.28)
Type of study										
Case-control	1.10 (0.86–1.41)	0.74	0.80 (0.78–0.83)	0.54	1.08 (0.94–1.25)	0.44	1.91 (1.79–2.03)	**<0.01**	1.10 (0.90–1.35)	0.75
Cohort	1.39 (1.17–1.64)		0.99 (0.74–1.33)		1.41 (1.20–1.65)		2.00 (1.16–3.48)		1.10 (0.89–1.36)	
RCT	0.90 (0.73–1.12)		–		0.95 (0.67–1.34)		–		0.99 (0.61–1.60)	
Events										
≥1000	1.22 (1.09–2.36)	0.77	1.04 (0.66–1.66)	0.58	1.23 (0.96–1.57)	0.98	1.76 (1.51–2.05)	0.73	1.06 (0.85–1.33)	0.92
<1000	1.31 (0.97–1.76)		0.93 (0.70–1.23)		1.21 (1.01–1.46)		2.36 (0.43–13.01)		1.09 (0.83–1.43)	
Type of glucocorticoid										
Inhaled	0.82 (0.69–0.97)	**0**.**01**	0.80 (0.77–0.82)	**<0.01**	0.97 (0.83–1.15)	**0**.**01**	–		0.99 (0.61–1.60)	0.63
Not Inhaled^a^	1.38 (1.25–1.51)		1.62 (1.27–2.07)		1.40 (1.20–1.63)		–		1.16 (0.97–1.39)	
Location										
Asia	0.44 (0.13–1.47)	0.76	–		2.56 (0.46–14.40)	0.16	1.79 (1.03–3.11)	0.40	0.75 (0.51–1.11)	0.06
Europe	1.44 (1.18–1.76)		1.13 (0.68–1.91)	0.16	1.33 (1.13–1.56)		2.32 (1.74–3.08)		1.10 (0.92–1.32)	
North American	1.22 (1.02–1.46)		0.85 (0.76–0.95)		1.13 (0.87–1.46)		0.99 (0.64–1.53)		1.30 (0.91–1.86)	
Baseline disease										
Rheumatoid arthritis	1.47 (1.20–1.80)	0.48	1.53 (1.11–2.11)	0.48	1.42 (1.21–1.68)	0.94	1.16 (0.63–2.14)	0.60	1.63 (1.20–2.20)	**0**.**01**
Chronic obstructive pulmonary disease/ Asthma	0.87 (0.68–1.11)		0.80 (0.78–0.82)		0.97 (0.76–1.24)		–		0.99 (0.78–1.25)	
Other^b^	1.31 (0.87–1.96)		1.34 (0.96–1.88)		1.41 (0.99–2.02)		2.72 (0.57–13.02)		0.94 (0.65–1.38)	
Age										
≥65 years	1.06 (0.88–1.27)	0.07	0.82 (0.76–0.89)	**<0.01**	1.09 (0.91–1.30)	0.09	1.35 (0.62–2.97)	**0**.**01**	1.00 (0.85–1.18)	0.09
<65 years	1.42 (1.16–1.75)		1.33 (0.78–2.26)		1.41 (1.23–1.61)		2.55 (1.14–5.65)		1.23 (1.02–1.49)	
Male proportion										
≥50%	0.81 (0.74–0.89)	**0**.**03**	0.92 (0.76–1.11)	0.67	0.90 (0.74–1.10)	**<0.01**	–		0.82 (0.61–1.10)	0.08
<50%	1.25 (1.13–1.39)		1.03 (0.67–1.60)		1.47 (1.27–1.69)		–		1.16 (0.99–1.37)	
Time periods of glucocorticoid use										
Current	1.38 (1.27–1.51)	0.84	1.04 (0.81–1.33)	0.41	1.39 (1.09–1.79)	0.16	2.04 (1.79–2.32)	**<0.01**	1.13 (0.91–1.41)	0.89
Ever	1.33 (0.93–1.88)		1.30 (0.56–3.02)		1.04 (0.76–1.43)		1.46 (1.18–1.81)		1.16 (0.83–1.63)	
Adjusted for risk profifiles										
No	1.06 (0.81–1.39)	0.23	0.69 (0.44–1.09)	0.16	1.36 (1.01–1.83)	0.56	1.25 (0.60–2.59)	0.37	0.89 (0.70–1.13)	0.11
Yes	1.34 (1.20–1.50)		1.04 (0.77–1.40)		1.22 (1.05–1.41)		2.14 (1.64–2.79)		1.17 (0.99–1.39)	
Publication year										
≥2010	1.41 (1.21–1.64)	0.06	1.35 (0.86–2.12)	**<0.01**	1.41 (1.22–1.64)	**0**.**02**	2.80 (1.30–6.02)	**0**.**02**	1.12 (0.96–1.31)	0.66
<2010	0.99 (0.76–1.30)		0.98 (0.76–1.25)		1.03 (0.89–1.41)		1.32 (0.77–2.28)		1.05 (0.68–1.63)	

MACE: major adverse cardiovascular events; CHD: coronary heart disease.

^a^ included oral, intramuscular, intravenous, rectal and so on.

^b^ included prostate cancer, pneumonia, skin disorders, inflammatory bowel disease, polymyalgia rheumatica, psoriatic arthritis and so on.

The bold words to emphasize that *p*-value less than 0.05.

**Figure 2:**
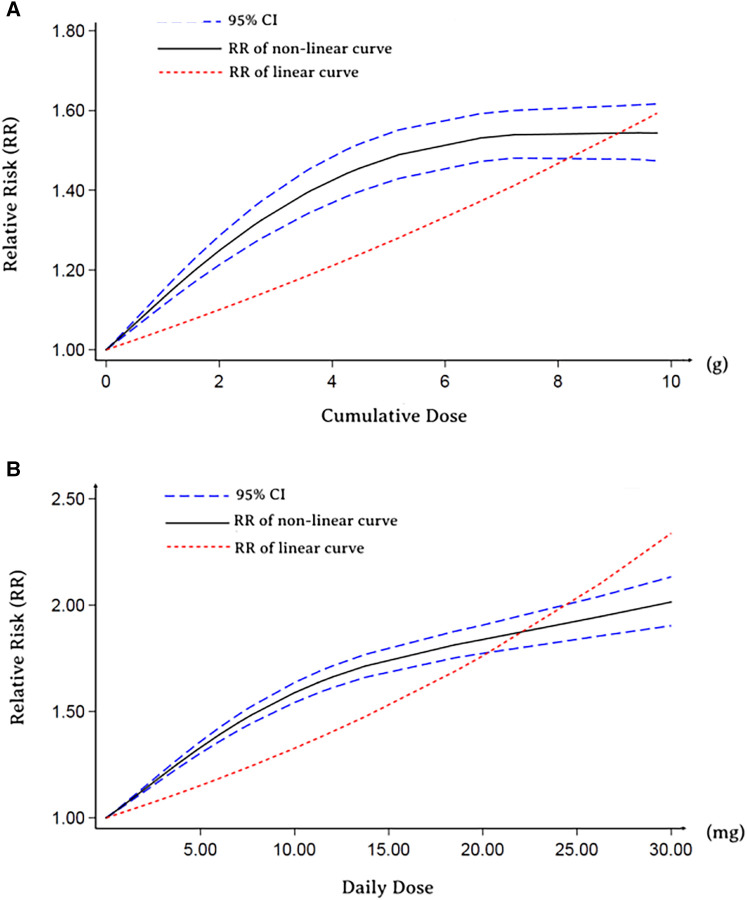
Dose-response relationship between RR of MACE and GCs. (**A**) Cumulative dose-response relationship between RR of MACE and GCs. (**B**) Daily dose-response relationship between RR of MACE and GCs.

Furthermore, we performed an analysis to assess the correlation between cumulative and the daily dose of GCs use and MACE. The types of GCs included in the dose-response analysis were not inhaled (most were oral), and the dosage was converted into a prednisolone-equivalent dose (Supplementary Table S3). As depicted in Figure 2A, both nonlinear dose-response and linear dose-response analyses revealed that the risk of MACE increased with increasing GCs cumulative dose. In addition, in the linear dose-response analysis, we concluded that the MACE risk increased by 10% (95% CI: 6%–15%) for each additional gram of GCs cumulative dose. A similar trend was observed in the analysis of the daily dose-response relation between GCs and MACE outcomes (Figure 2B). Likewise, the increment per 10 micrograms daily risk was estimated to be 63% (95% CI: 46%–83%).

### Association between GCs and coronary heart disease

**Figure 3:**
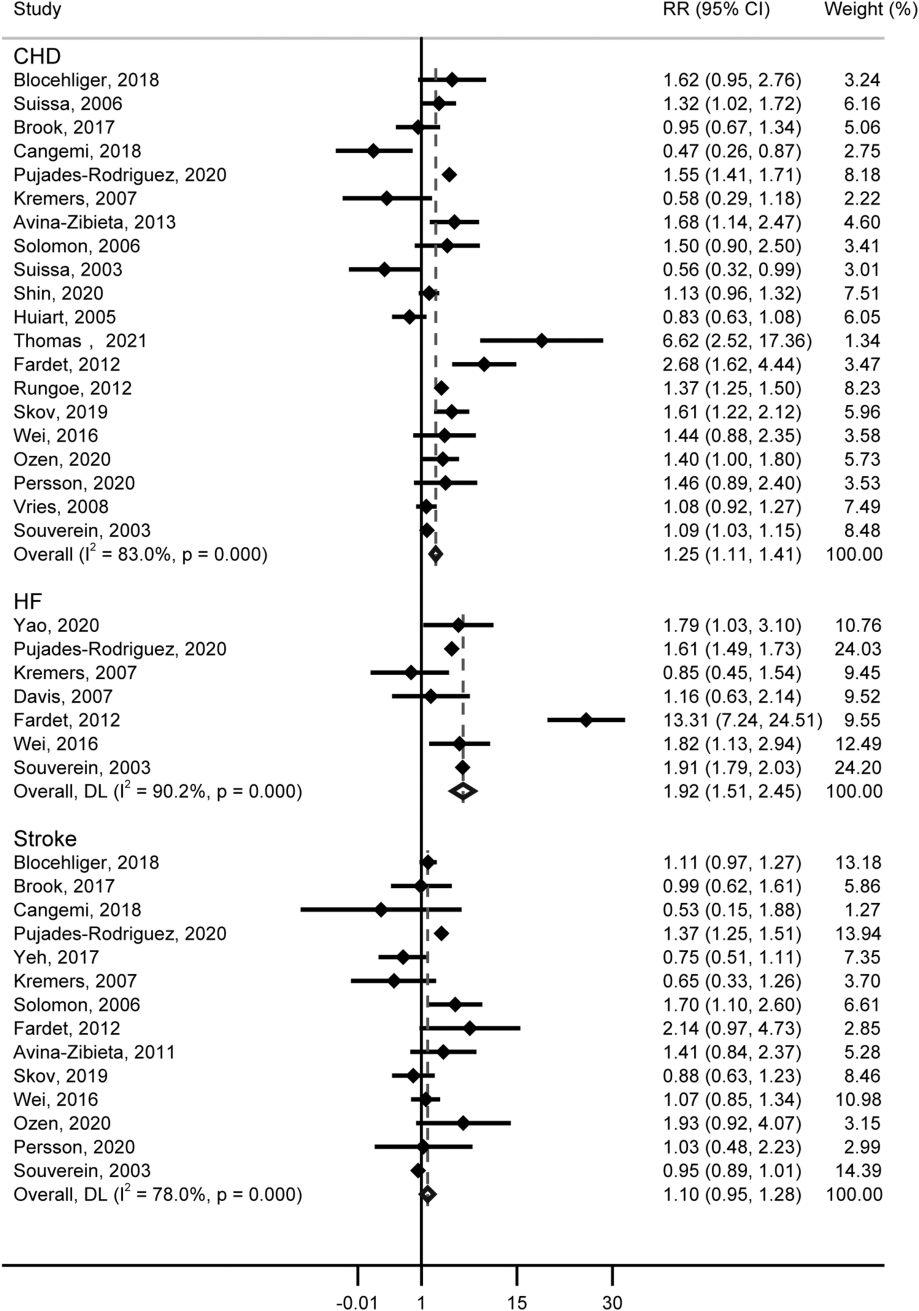
Forest plot showing the RR of CHD, HF and sroke.

For the risk of CHD, 20 studies (including 622,097 patients) contained information on GCs use, and GCs were associated with an increased risk of CHD (RR = 1.25, 95% CI: 1.11–1.41, Figure 3). Substantial heterogeneity was detected (Cochran’s *Q* = 111.56, *I*^2^ = 83.0%, *P* < 0.001), and the type of GCs, male proportion, and publication year showed potential sources of between-study heterogeneity (Table 1). Additionally, we calculate an absolute risk of CHD increase with 4.16 (95% CI: 2.58–5.74) cases per 1,000 person-years among GCs users. Neither the funnel plot (Supplementary Figure S2B) nor Egger (*P* = 0.586) and Begg (*P* = 0.922) tests showed publication bias.

### Association between GCs and heart failure

Seven studies about the relationship between GCs and HF contain data from 14,811,957 patients. A significantly increased risk of HF was observed, for GCs use (RR = 1.92, 95% CI: 1.51–2.45, Figure 3), with substantial heterogeneity (Cochran’s *Q* = 60.99, *I*^2 ^= 90.2%, *P* < 0.001). In studies included, the Yao’s study contains over 14 million patients, which represents over 90% of the subjects studied. Thus a separate analysis excluding this study was performed, and we also found that GCs were associated with an increased risk of HF (RR = 1.943, 95% CI: 1.50–2.52). In the subgroup analysis, we found the differences in the type of study, age, and time periods of GCs use would contribute to heterogeneity (Table 1). Further, the absolute risk of HF increased by 3.14 (95% CI: 3.38–3.51) cases per 1,000 person-years in GCs users. No publication bias was indicated (Funnel plot in Supplementary Figure S2C, Egger, *P* = 0.773, Begg, *P* = 0.548).

### Association between GCs and stroke

Figure 3 shows the results from the random effects model combining the RR for stroke, and no significant correlation was observed with GCs (RR = 1.10, 95% CI: 0.95–1.28). The heterogeneity analyses suggested moderate heterogeneity across these studies (Cochran’s *Q* = 59.00, *I*^2^ = 78.0%, *P* < 0.001). Differences in study type, events, GCs type, age, male proportion, time periods of GCs use, whether adjusted for risk profiles, and publication year were not potential sources of heterogeneity (Table 1). Funnel plot (Supplementary Figure S2D), Egger (*P* = 0.671), and Begg (*P* = 0.827) suggested an absence of publication bias.

### Association between GCs and all-cause death

**Figure 4:**
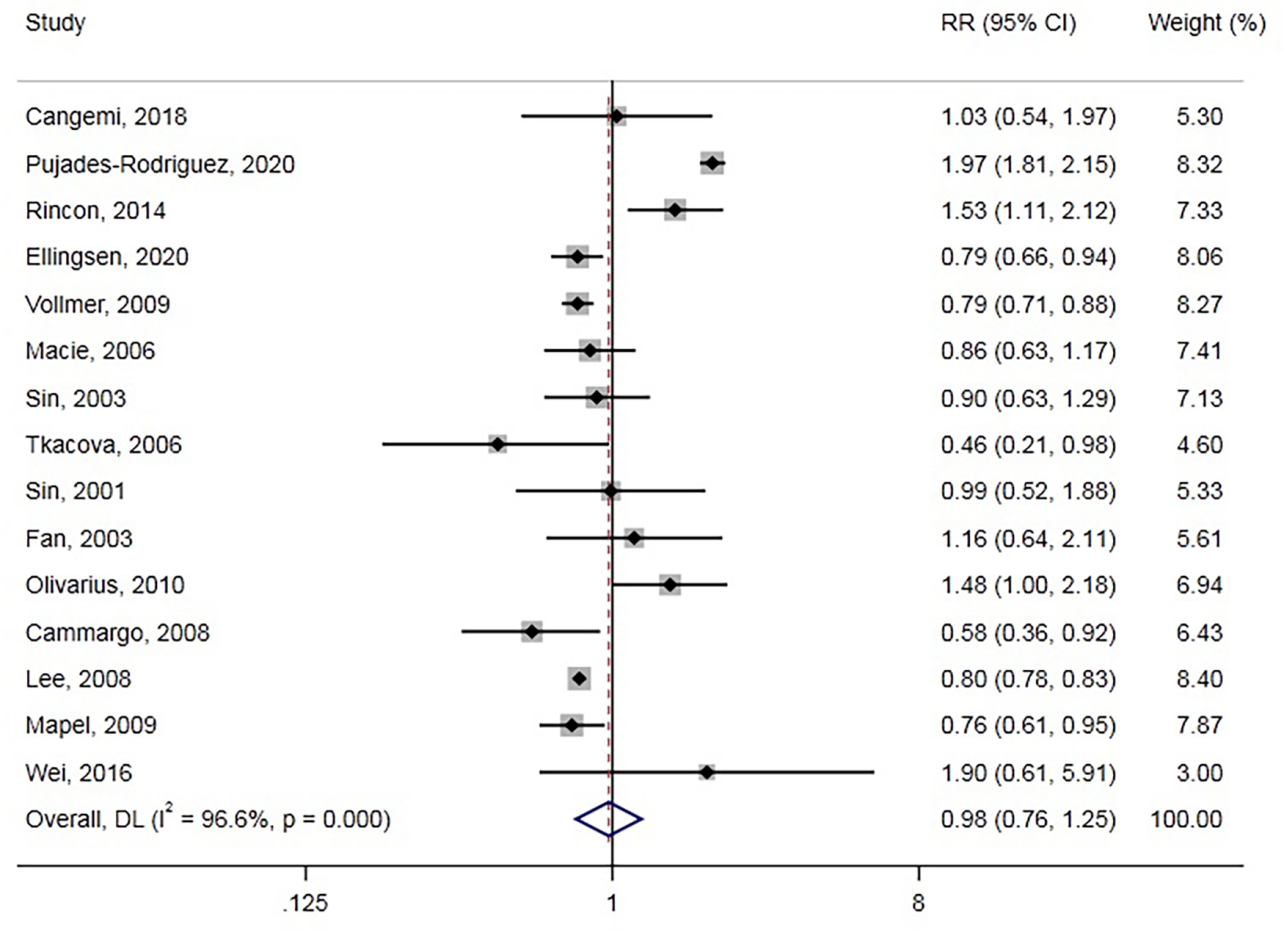
Forest plot showing the RR of all-cause death.

All-cause death was reported in 15 studies involving 661,256 participants. Overall, GC use was not associated with all-cause death (RR = 0.98, 95% CI: 0.76–1.25, Figure 4), with significant between-study heterogeneity (Cochran’s *Q* = 406.70, *I*^2^ = 96.6%, *P* < 0.001). We also conducted a subgroup analysis and meta-regression in different subgroups (Table 1). GCs type and population age could be sources of heterogeneity. Moreover, we found that inhaled GCs were associated with decreased risk of all-cause death (RR = 0.80, 95% CI: 0.77–0.82). Also, previous GCs use and current GCs use were not associated with all-cause death. Besides, GCs use decreased all-cause death risk in some subgroup analyses, including studies of case-control, North American, chronic obstructive pulmonary disease/asthma, and age >=65 years. The funnel plot (Supplementary Figure S2F) showed no evidence of publication bias with Egger, *P* = 0.452, Begg, *P* = 0.276.

## Discussion

Conflicting evidence exists of an association between GCs use and the risk of adverse cardiovascular events. A systematic review of the literature provided insight into this question. Based on our systematic review and meta-analysis, there were two major findings. First, the results of this study indicated that GCs were associated with increased risks of MACE, CHD, and HF. Second, the risk of MACE increased with increasing cumulative or daily doses of GCs.

Given that GCs are commonly used drugs in many countries, and millions of patients with inflammatory diseases are prescribed annually, it is difficult to identify the potential effects of GCs on cardiovascular events. However, the risk of composites of cardiovascular or CHD, and HF associated with the use of GC in patients has been assessed recently. In a population-based case-control study, Souverein found that ever use of oral GCs use was significantly associated with cardiovascular or cerebrovascular effects (adjusted OR 1.25, 95% CI: 1.21–1.29) (25). Similarly, patients requiring oral corticosteroids had a significantly higher risk of CHD, as observed by Rungoe (26). Additionally, a strong relationship between GCs and HF was reported in Yao’s study (27). These relationships may partly be explained by corticosteroid-inducing hypertension (28), one of the most critical risk factors for CVDs. Besides, exposure to corticosteroids was known to increase the risk of developing type 2 diabetes (29) which may also increase the risk of CVDs. Another possible explanation for this was that GCs would lead to lipodystrophy (30) and promote the reabsorption of water and sodium in the kidneys (31). Further, in addition to glucocorticoid receptors, some of GCs (e.g., hydrocortisone, prednisone) also act by way of mineralocorticoid receptors (32). It is widely known that there is an association between an increase in mineralocorticoid-receptor activation and hypertension and cardiovascular aging (33). Moreover, GCs vary in their mineralocorticoid effects, but it is a pity that there were insufficient data to perform a sub-analysis looking at the primary outcomes stratified by mineralocorticoid effect.

Contrary to expectations, this study did not find a significant difference between GCs and stroke, although there were shared many risk factors contributing to the occurrence of both stroke and CHD. It is well established that GCs were available to act on the vascular wall, endothelial, and vascular smooth muscle (34). Also, brain microvascular endothelium was found to benefit from GC action by tightening the barrier in a vitro study (35). Consequently, the different results between CHD and stroke could be attributed to the difference in GC receptor status and GC responsivity between endothelial cells from the brain and the heart (36).

In addition, this study’s observed difference between GCs and all-cause death was insignificant. It could be due to the GCs’ anti-inflammatory effect being favorable for some participants’ survival though GCs increased MACE risk. In a previous study, The Lung Health Study Research Group demonstrated that corticosteroids might attenuate airway hyperresponsiveness (37), which was a known risk mortality factor in COPD (38).

The results suggested that the association of GCs with MACE risk may be mediated by an acute mechanism for the higher risk in current compared to ever GCs users. Some plausible explanations have been proposed. First, Börcsök et al. reported that plasma endothelin-1 (a vasoconstrictor associated with cardiovascular disease) level increased 50% early in human subjects after prednisolone use (39). Second, because glucocorticoids suppress the immune system, patients who receive them are more susceptible to infection, which is one of the most common causes of thrombosis (40, 41). Unsurprisingly, it was found that decreased exposure to glucocorticoids translated into a reduction in the risk of invasive fungal infection and Gram-positive bacteremia (42). Similarly, glucocorticoid avoidance and withdrawal could lead to the reduction of cardiovascular risk factors (43). These findings might suggest an acute causal association between MACE and GCs.

Notably, based on the subgroup analysis, inhaled GCs were associated with a reduction in MACE and all-cause death without increasing the risk of CHD or stroke. The mechanisms that underlie these responses, however, are poorly defined. As mentioned above, the risk of MACE increased with increasing GCs cumulative dose. It is possible that harmful systemic reactions were avoided because very little of GCs could become systemic when GCs were administered topically, bringing substantial benefit. Sin et al. reported that ICS therapy reduced C-reactive protein (CRP) levels by 50% after two weeks (44). Thus, ICS might exert cardioprotective effects by reducing the transcription of CRP, which has been shown to be one of the independent predictors of cardiac events (45).

Our findings have important medical implications. It remains unclear whether GCs cause adverse cardiac events, and the risks of GC use have attracted much attention among clinicians and patients. Our research timely evaluates the evidence of suspected risks. The increased risk of CVDs, including MACE, CHD, and HF, was observed in our study, which supported close monitoring of CVDs is warranted in patients during GCs treatment. Also, clinicians should be mindful that regular use of high-dose GCs should be avoided where possible, and decrement and discontinuance in GCs should be early. In addition, inhaled GCs seem to be safe in patients.

Our study has several strengths, including the strict criteria of inclusion, the large sample size, the diversity of the study population, the dose-response relationship between GCs and MACE, and pre-specified subgroup analyses. Besides, the robustness of the study findings is supported by the absence of important publication bias.

## Limitations

Nevertheless, several limitations of our study were also discussed. First, the study has its inherent limitations of being a retrospective analysis. Second, there is substantial heterogeneity between the different studies. The therapeutic protocols of GCs were not equivalent in different studies. Population characteristics, including age, sex, presence or absence of hypertension, diabetes, and dyslipidaemia might also introduce heterogeneity into studies. And as only one study in this literature search is a randomized-controlled trial, the data are susceptible to indication bias. Third, adjusted hazard ratios were not used in some included studies, which may also influence outcomes. Finally, comprehensive individual patient data were unavailable from the studies, we were thus unable to identify the independent associations of individual variables with study outcomes. Such as, the conclusions regarding GCs dosing would be influenced as it is not clear if the GCs were dosed based on weight, severity of illness (i.e., escalating doses), clinical guidelines/local protocols and this will likely be impossible to fully investigate with this meta-analysis approach. Furthermore, the lack of individual patient data limited further analysis such as meta-regression.

## Conclusions

In conclusion, the results of this meta-analysis reveal that GCs is possibly related with increased risk for MACE, CHD, and HF but not all-cause death or stroke. The dose-response analysis highlighted the risk of MACE may increased with increasing GCs cumulative or daily dose was observed. In addition, it seems that not inhaled GCs, and current GCs use were associated with higher risk. The association between GCs and CVDs has clinical relevance with respect to individual screening and the prevention of CVDs. This calls for further large RCTs warranted to confirm these findings and, additional studies will be needed to clarify the underlying mechanism.

## Data Availability

The original contributions presented in the study are included in the article/**Supplementary Material**, further inquiries can be directed to the corresponding authors.

## Supplementary material

SUPPLEMENTARY FIGURE S1

Flowchart of the selection of studies included in meta-analysis.

SUPPLEMENTARY FIGURE S2

Funnel plots showing association of cardiovascular risk with GCs.

SUPPLEMENTARY TABLE S1

Characteristics of the included studies.
