# Supplementary figures and images for: The role of glucocorticoids in increasing cardiovascular risk
Source: Front Cardiovasc Med. 2023 Jul 5;10:1187100. doi: 10.3389/fcvm.2023.1187100 (PMC10354523; doi:10.3389/fcvm.2023.1187100)

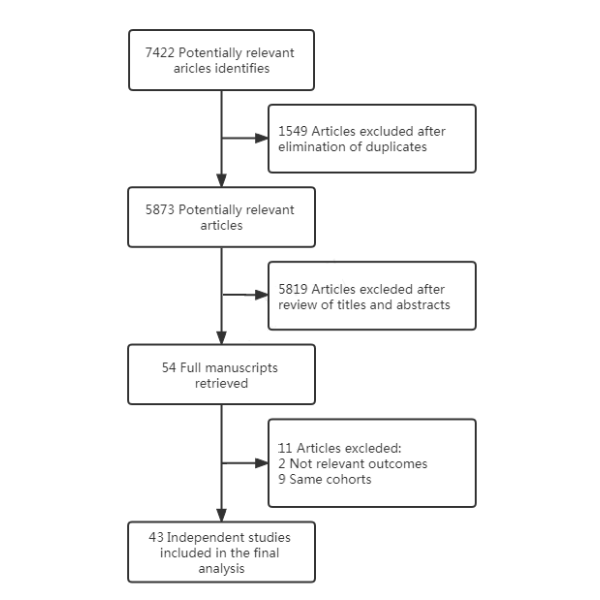

Supplement: Supplementary file 1 [file Image1.tiff]

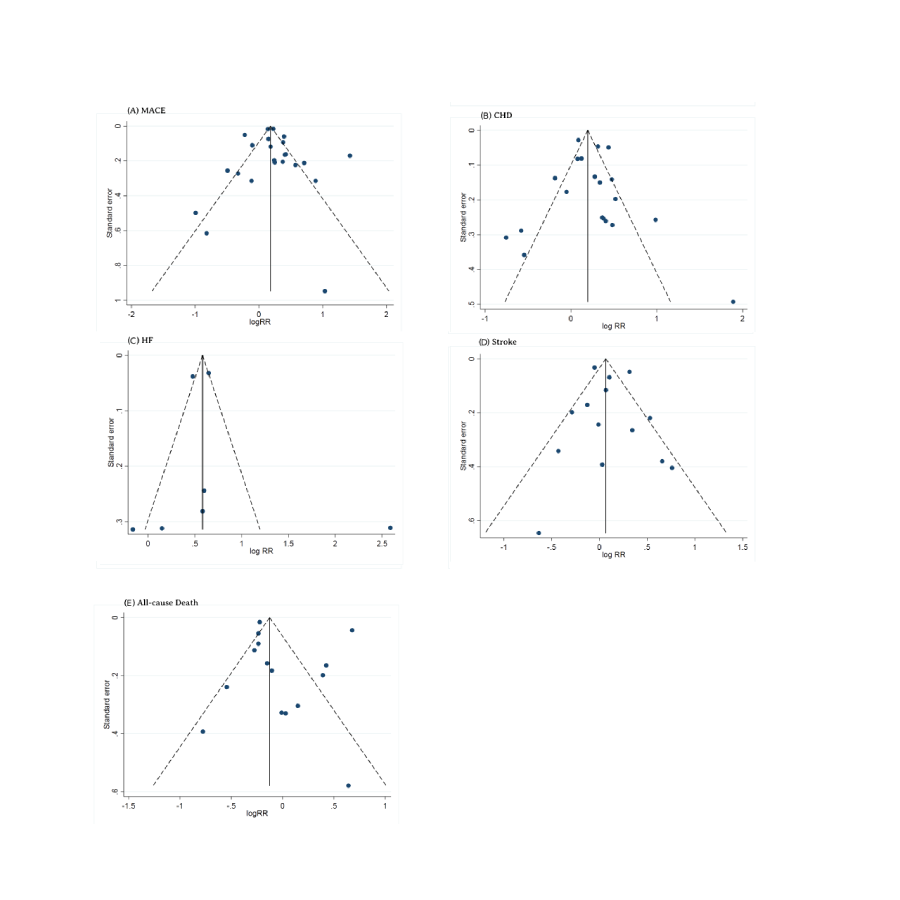

Supplement: Supplementary file 2 [file Image2.tiff]
