# Supplementary material for: The role of glucocorticoids in increasing cardiovascular risk
Source: Front Cardiovasc Med. 2023 Jul 5;10:1187100. doi: 10.3389/fcvm.2023.1187100 (PMC10354523; doi:10.3389/fcvm.2023.1187100)
Supplement: Supplementary file 3 [file Table1.docx]

| **Study** | **Publication year** | **Source of Participants** | **Region** | **Type of Study** | **Baseline Diseases** | **Follow-up (year)** | **Simple Size** | **Type of Glucocorticoid** | **Cumulative Dose of Glucocorticoid** | **Daily Dose of Glucocorticoid** | **Outcomes** |
| --- | --- | --- | --- | --- | --- | --- | --- | --- | --- | --- | --- |
| Schultz, 2019(1) | 2019 | Truven Health Analytics MarketScan Commercial Claims and Encounters Database and Medicare Supplemental Database | USA | Population-based cohort | Castration-resistant prostate cancer | 1 | 9425 | Oral betamethasone, budesonide, cortisone, dexamethasone, hydrocortisone, methylprednisolone, prednisolone, prednisone, and triamcinolone | （prednisone equivalent） low ＜ 0.5g, medium 0.5-2.0g, high ＞ 2.0g | low ＜ 1.4mg, medium 1.4–5.6mg, high ＞5.6mg, | MACE |
| Blocehliger, 2018(2) | 2018 | Clinical Practice Research Datalink | UK | Case-control study | Asthma | N/A | 19959 | Oral  prednisolone | low ＜ 0.5g, medium 0.5–2.0g, high ＞ 2.0g | low ≤ 1mg, medium 1–5 mg, high ＞ 5mg | MACE, coronary heart disease, stroke |
| Suissa, 2006(3) | 2006 | PharMetrics Patient-Centric Outcomes Database | North American | Case-control study | Rheumatoid arthritis | 1.2 | 6138 | nonspecific | N/A | N/A | Coronary heart disease |
| Yao, 2020(4) | 2020 | National Health Insurance Research Database in Taiwan | China | Population-based cohort | Skin disorders, respiratory tract infections | 0.25 | 14467072 | Oral nonspecific | N/A | 10mg | Heart failure |
| Innala, 2011(5) | 2011 | Swedish Rheumatoid Arthritis Registry | Sweden | Population-based cohort | Rheumatoid arthritis | 5 | 442 | nonspecific | N/A | N/A | MACE |
| Brook, 2017(6) | 2017 | SUMMIT trial | 43 countries | Randomized controlled trial | Chronic obstructive pulmonary disease | 3 | 8246 | Inhaled fluticasone furoate | N/A | 100ug | MACE, coronary heart disease, stroke |
| Cangemi, 2019(7) | 2019 | University Hospital Policlinico Umberto I | Italy | Population-based cohort | Pneumonia | 0.1 | 758 | Oral or intravenous methylprednisolone, betamethasone, prednisone | N/A | methylprednisolone (60%; 20–80mg), betamethasone (22%; 4–8mg), and prednisone (18%; 25–50mg). | MACE, all-cause death, coronary heart disease, stroke |
| Halm, 2006(8) | 2006 | Jan van Breemen Institute | Netherlands | Case-control study | Rheumatoid arthritis | N/A | 613 | Oral prednisone | N/A | N/A | MACE |
| Pujades-Rodriguez, 2020(9) | 2020 | Clinical Practice Research Datalink | UK | Population-based cohort | immune-mediated inflammatory disease(polymyalgia and/or giant cell arteritis, inflammatory bowel disease, rheumatoid arthritis, vasculitis, and systemic lupus erythematosus) | 5.9 | 87794 | Oral nonspecific | (prednisolone equivalent) 1–959.9mg, 960–3054.9mg, 3055–7299.9mg, ≥ 7300mg | 1–4.9mg, 5.0–14.9mg 1.89, 15.0–24.9mg 2.38, ≥25mg | MACE, all-cause death, coronary heart disease, stroke |
| Yeh, 2017(10) | 2017 | Taiwan’s Longitudinal Health Insurance Database 2000 | China | Population-based cohort | Asthma–chronic obstructive pulmonary disease syndrome | N/A | 10260 | Oral nonspecific Inhaled fluticasone propionate and budesonide | N/A | N/A | Stroke |
| Rincon, 2014(11) | 2014 | 6 local rheumatology clinics | USA | Population-based cohort | Rheumatoid arthritis | 9.2 | 779 | nonspecific | (prednisone equivalent) ＜ 9g,  9–39.9g,  ≥ 40g | ＜5mg, 5-7mg, 8–15mg, ≥15mg | MACE, all-cause death |
| Kremers, 2007(12) | 2007 | Rochester Epidemiology Project | USA | Population-based cohort | Polymyalgia rheumatica | 7.6 | 364 | Oral or parenteral nonspecific | (prednisone equivalent) low ≤ 35mg, medium ＞ 35 to ≤ 3000mg, high ＞ 3000mg | N/A | MACE, coronary heart disease, heart failure, stroke |
| Davis, 2007(13) | 2007 | Rochester Epidemiology Project | USA | Population-based cohort | Rheumatoid arthritis | 15 | 603 | Oral or parenteral nonspecific | (prednisone equivalent) low ≤ 1500mg, mid ＞ 1500 to ≤ 7000mg, high ＞ 7000mg | ≤ 7.5mg, ＞ 7.5mg | MACE, heart failure |
| Gonzalez-Gay, 2007(14) | 2007 | Hospital Xeral-Calde, Lugo | Spain | Population-based cohort | Rheumatoid arthritis | 13.4 | 182 | nonspecific prednisone | N/A | N/A | MACE |
| Avina-Zibieta, 2013(15) | 2013 | British Columbia Ministry of Health | Canada | Population-based cohort | Rheumatoid arthritis | 6 | 8384 | Oral nonspecific | N/A | N/A | Coronary heart disease |
| Solomon, 2006(16) | 2006 | Medicare enrollees who were also beneficiaries in the Pharmaceutical Assistance Contract for the Elderly | USA | Case-control study | Rheumatoid arthritis | N/A | 7550 | Oral nonspecific | N/A | N/A | MACE, coronary heart disease, stroke |
| Ellingsen, 2020(17) | 2020 | Primary healthcare centers | Sweden | Population-based cohort | Chronic obstructive pulmonary disease | 3.6 | 16251 | Inhaled nonspecific | N/A | N/A | All-cause death |
| Vollmer, 2009(18) | 2009 | KPNW’s electronic medical record | USA | Population-based cohort | Chronic obstructive pulmonary disease | 3.15 | 2902 | Inhaled nonspecific | N/A | N/A | All-cause death |
| Suissa, 2003(19) | 2003 | Databases of Saskatchewan Health | Canada | Case-control study | Asthma | N/A | 1038 | Inhaled nonspecific | N/A | N/A | Coronary heart disease |
| Macie, 2006(20) | 2006 | Manitoba Population Health Research Repository | Canada | Population-based cohort | Chronic obstructive pulmonary disease | 1 | 4987 | Oral or inhaled nonspecific | N/A | N/A | All-cause death |
| Sin, 2003(21) | 2003 | Canadian Institute for Health Information, Alberta | Canada | Population-based cohort | Chronic obstructive pulmonary disease | 2.64 | 6740 | Oral or inhaled nonspecific | N/A | N/A | All-cause death |
| Tkacova, 2006(22) | 2006 | Department of Respiratory Medicine and Tuberculosis of the Teaching Hospital | Slovakia | Population-based cohort | Chronic obstructive pulmonary disease | 1 | 145 | Inhaled nonspecific | N/A | N/A | All-cause death |
| Sin, 2001(23) | 2001 | Canadian Institute for Health Information, Ontario | Canada | Population-based cohort | Chronic obstructive pulmonary disease | 1 | 22620 | Oral or inhaled nonspecific | N/A | N/A | All-cause death |
| Fan, 2003(24) | 2003 | Ambulatory Care Quality Improvement Project(ACQUIP) trial | USA | Population-based cohort | Chronic obstructive pulmonary disease | 1.5 | 8052 | Oral prednisone inhaled triamcinolone, beclomethasone, flunisolide, fluticasone | N/A | N/A | All-cause death |
| Shin, 2020(25) | 2020 | Korean National Health Insurance Service-National Sample Cohort | Korea | Population-based cohort | Chronic obstructive pulmonary disease | 3.3 | 4400 | Inhaled beclomethasone, budesonide, ciclesonide, flunisolide, fluticasone, triamcinolone | （fluticasone equivalent） ＜ 30000ug, 30000-75125ug, 75125-207500ug, ＞ 207500ug | N/A | Coronary heart disease |
| Huiart, 2005(26) | 2005 | Saskatchewan health services databases | Canada | Case-control study | Chronic obstructive pulmonary disease | 1 | 2235 | Inhaled nonspecific | N/A | (beclomethasone equivalent) ≤ 50ug, 50-200ug, 200-500ug, ＞ 500ug | Coronary heart disease |
| Thomas， 2021(27) | 2021 | Clalit Health Services | Israel | Population-based cohort | Arthritis and other musculoskeletal conditions | 0.02 | 60856 | Intra-articular and soft-tissue nonspecific | N/A | N/A | Coronary heart disease |
| Olivarius, 2010(28) | 2010 | Diabetes Care in General Practice study | Denmark | Population-based cohort | Diabetes | 14 | 1369 | Oral  prednisolone or prednisone | N/A | N/A | All-cause death |
| Cammargo, 2008(29) | 2008 | Nurses' Health Study | USA | Population-based cohort | Asthma | 5 | 2671 | Inhaled nonspecific | N/A | N/A | All-cause death |
| Yeh, 2022(30) | 2022 | Longitudinal Health Insurance Database 2000 | China | Population-based cohort | Bronchiectasis-asthma combination | N/A | 1706 | Oral or inhaled nonspecific | N/A | N/A | MACE |
| Lee, 2008(31) | 2008 | National Veterans Affairs inpatient, outpatient, pharmacy, and mortality databases; Centers for Medicare & Medicaid Services databases; and National Death Index Plus data | USA | Case-control study | Chronic obstructive pulmonary disease | N/A | 352631 | Inhaled nonspecific | N/A | N/A | MACE, all-cause death |
| Fardet, 2012(32) | 2012 | Health Improvement Network | UK | Population-based cohort | Iatrogenic Cushing’s syndrome | 1 | 3829 | Oral, intramuscular or intravenous prednisolone, prednisone, dexamethasone, triamcinolone, betamethasone, methylprednisolone, and deflazacort | N/A | N/A | MACE, coronary heart disease, heart failure, stroke |
| Avina-Zibieta, 2011(33) | 2011 | British Columbia Ministry of Health | Canada | Population-based cohort | Rheumatoid arthritis | 6 | 7051 | Oral nonspecific | N/A | N/A | Stroke |
| Rungoe, 2012(34) | 2012 | Danish National Patient Register | Denmark | Population-based cohort | Inflammatory bowel disease | 11.3 | 28833 | Oral nonspecific | N/A | N/A | Coronary heart disease |
| Skov, 2019(35) | 2019 | Swedish National Patient Register | Sweden | Population-based cohort | Autoimmune Addison disease | 7.5 | 15258 | Hydrocortisone, cortisone acetate | N/A | (Hydrocortisone equivalent) low 19.2mg, intermiate 29.4mg, high 40.3mg | MACE, coronary heart disease, stroke |
| Ocon , 2021(36) | 2021 | CorEvitas RA registry | USA | Population-based cohort | Rheumatoid arthritis | 3.3 | 19902 | nonspecific | (prednisone equivalent) 1-500mg, 501-1100mg, 1101-2100mg, ＞2100mg | 1-＜5mg, ≥5-9mg,  ≥10mg | MACE |
| Mapel, 2009(37) | 2009 | Administrative databases of four integrated healthcare delivery systems | USA | Population-based cohort | Chronic obstructive pulmonary disease | N/A | 2574 | Inhaled nonspecific | N/A | N/A | All-cause death |
| Wei, 2016(38) | 2016 | MEMO record linkage database | UK | Population-based cohort | Different disease | 3.1 | 150983 | inhaled, topical, oral and parenteral, rectal application steroids, nasal nonspecific | N/A | N/A | MACE, all-cause death, coronary heart disease, heart failure, stroke |
| Ozen, 2020(39) | 2020 | FORWARD, the National Databank for Rheumatic Diseases longitudinal prospective observational study | USA | Population-based cohort | Rheumatoid arthritis | 5.1 | 18754 | Oral nonspecific | N/A | (prednisolone equivalent) ＜ 7.5mg ≥ 7.5mg | MACE, coronary heart disease, stroke |
| Persson, 2020(40) | 2020 | United States MarketScan database | USA | Population-based cohort | Psoriatic arthritis | 1.9 | 68678 | Oral or injection nonspecific | N/A | N/A | MACE, coronary heart disease, stroke |
| Greenberg, 2010(41) | 2010 | Consortium of Rheumatology Researchers of North America RA registry | USA | Population-based cohort | Rheumatoid arthritis | 1.9 | 10156 | Oral  prednisolone | N/A | ＜ 7.5mg, ≥ 7.5mg | MACE |
| Vries, 2008(42) | 2008 | PHARMO record linkage system | Netherlands | Case-control study | N/A | N/A | 26728 | Inhaled nonspecific | N/A | (beclomethasone equivalent) ≤400ug, 401-800ug,  801-1600ug, ≥1600ug | Coronary heart disease |
| Souverein, 2003(43) | 2003 | General practice research database | UK | Case-control study | Rheumatoid arthritis, Chronic obstructive pulmonary disease, other | N/A | 101312 | Oral  cortisone, hydrocortisone, prednisone, prednisolone, triamcinolone, methylprednisone, dexamethasone, and betamethasone | N/A | N/A | MACE, coronary heart disease, heart failure, stroke |

**References:**

1. Schultz NM, Penson DF, Wilson S, et al. Adverse Events Associated with Cumulative Corticosteroid Use in Patients with Castration-Resistant Prostate Cancer: An Administrative Claims Analysis. Drug Safety 2020;43:23-33.

2. Bloechliger M, Reinau D, Spoendlin J, et al. Adverse events profile of oral corticosteroids among asthma patients in the UK: cohort study with a nested case-control analysis. Respiratory Research 2018;19.

3. Suissa S, Bernatsky S, Hudson M. Antirheumatic drug use and the risk of acute myocardial infarction. Arthritis & Rheumatism 2006;55:531-536.

4. Yao TC, Huang YW, Chang SM, Tsai SY, Wu AC, Tsai HJ. Association Between Oral Corticosteroid Bursts and Severe Adverse Events : A Nationwide Population-Based Cohort Study. Ann Intern Med 2020;173:325-330.

5. Innala L, Moller B, Ljung L, et al. Cardiovascular events in early RA are a result of inflammatory burden and traditional risk factors: a five year prospective study. Arthritis Res Ther 2011;13:R131.

6. Brook RD, Anderson JA, Calverley PM, et al. Cardiovascular outcomes with an inhaled beta2-agonist/corticosteroid in patients with COPD at high cardiovascular risk. Heart 2017;103:1536-1542.

7. Cangemi R, Falcone M, Taliani G, et al. Corticosteroid Use and Incident Myocardial Infarction in Adults Hospitalized for Community-acquired Pneumonia. Ann Am Thorac Soc 2019;16:91-98.

8. van Halm VP, Nurmohamed MT, Twisk JW, Dijkmans BA, Voskuyl AE. Disease-modifying antirheumatic drugs are associated with a reduced risk for cardiovascular disease in patients with rheumatoid arthritis: a case control study. Arthritis Res Ther 2006;8:R151.

9. Pujades-Rodriguez M, Morgan AW, Cubbon RM, Wu J. Dose-dependent oral glucocorticoid cardiovascular risks in people with immune-mediated inflammatory diseases: A population-based cohort study. PLOS Medicine 2020;17:e1003432.

10. Yeh JJ, Wei YF, Lin CL, Hsu WH. Effect of the asthma-chronic obstructive pulmonary disease syndrome on the stroke, Parkinson's disease, and dementia: a national cohort study. Oncotarget 2018;9:12418-12431.

11. Del Rincón I, Battafarano DF, Restrepo JF, Erikson JM, Escalante A. Glucocorticoid Dose Thresholds Associated With All-Cause and Cardiovascular Mortality in Rheumatoid Arthritis. Arthritis & Rheumatology 2014;66:264-272.

12. Kremers HM, Reinalda MS, Crowson CS, Davis JM, Hunder GG, Gabriel SE. Glucocorticoids and cardiovascular and cerebrovascular events in polymyalgia rheumatica. Arthritis & Rheumatism 2007;57:279-286.

13. Davis JM, Maradit Kremers H, Crowson CS, et al. Glucocorticoids and cardiovascular events in rheumatoid arthritis: A population-based cohort study. Arthritis & Rheumatism 2007;56:820-830.

14. Gonzalez-Gay MA, Gonzalez-Juanatey C, Lopez-Diaz MJ, et al. HLA–DRB1 and persistent chronic inflammation contribute to cardiovascular events and cardiovascular mortality in patients with rheumatoid arthritis. Arthritis & Rheumatism 2007;57:125-132.

15. Avina-Zubieta JA, Abrahamowicz M, De Vera MA, et al. Immediate and past cumulative effects of oral glucocorticoids on the risk of acute myocardial infarction in rheumatoid arthritis: a population-based study. Rheumatology 2012;52:68-75.

16. Solomon DH, Avorn J, Katz JN, et al. Immunosuppressive medications and hospitalization for cardiovascular events in patients with rheumatoid arthritis. Arthritis & Rheumatism 2006;54:3790-3798.

17. Ellingsen J, Johansson G, Larsson K, et al. <p>Impact of Comorbidities and Commonly Used Drugs on Mortality in COPD – Real-World Data from a Primary Care Setting</p>. 2020;Volume 15:235-245.

18. Vollmer WM, Peters D, Crane B, Kelleher C, Buist AS. Impact of regular inhaled corticosteroid use on chronic obstructive pulmonary disease outcomes. COPD 2007;4:135-42.

19. Suissa S, Assimes T, Brassard P, Ernst P. Inhaled corticosteroid use in asthma and the prevention of myocardial infarction. The American Journal of Medicine 2003;115:377-381.

20. Macie C, Wooldrage K, Manfreda J, Anthonisen NR. Inhaled corticosteroids and mortality in COPD. Chest 2006;130:640-6.

21. Sin DD, Man SFP. Inhaled corticosteroids and survival in chronic obstructive pulmonary disease: does the dose matter? European Respiratory Journal 2003;21:260-266.

22. Tkacova R, Toth S, Sin DD. Inhaled corticosteroids and survival in COPD patients receiving long-term home oxygen therapy. Respiratory Medicine 2006;100:385-392.

23. SIN DOND, TU JV. Inhaled corticosteroids and the risk of mortality and readmission in elderly patients with chronic obstructive pulmonary disease. American journal of respiratory and critical care medicine 2001;164:580-584.

24. Fan VS, Bryson CL, Curtis JR, et al. Inhaled Corticosteroids in Chronic Obstructive Pulmonary Disease and Risk of Death and Hospitalization. American Journal of Respiratory and Critical Care Medicine 2003;168:1488-1494.

25. Shin J, Yoon H, Lee YM, Ha E, Lee JH. Inhaled corticosteroids in COPD and the risk for coronary heart disease: a nationwide cohort study. Scientific Reports 2020;10.

26. Huiart L. Low-dose inhaled corticosteroids and the risk of acute myocardial infarction in COPD. European Respiratory Journal 2005;25:634-639.

27. Thomas K, Schonmann Y. Orthopaedic corticosteroid injections and risk of acute coronary syndrome: a cohort study. British Journal of General Practice 2021;71:e128-e133.

28. Olivarius NDF, Siersma V, Dyring-Andersen B, Drivsholm T, Hansen LJ, Henriksen JE. Patients Newly Diagnosed with Clinical Type 2 Diabetes during Oral Glucocorticoid Treatment and Observed for 14 Years: All-Cause Mortality and Clinical Developments. Basic & Clinical Pharmacology & Toxicology 2011;108:285-288.

29. Camargo CA, Barr RG, Chen R, Speizer FE. Prospective study of inhaled corticosteroid use, cardiovascular mortality, and all-cause mortality in asthmatic women. Chest 2008;134:546-551.

30. Yeh J, Lai M, Yang Y, Hsu C, Kao C. Relationships Between Bronchodilators, Steroids, Antiarrhythmic Drugs, Antidepressants, and Benzodiazepines and Heart Disease and Ischemic Stroke in Patients With Predominant Bronchiectasis and Asthma. Frontiers in Cardiovascular Medicine 2022;9.

31. Lee TA, Pickard AS, Au DH, Bartle B, Weiss KB. Risk for death associated with medications for recently diagnosed chronic obstructive pulmonary disease. Ann Intern Med 2008;149:380-90.

32. Fardet L, Petersen I, Nazareth I. Risk of cardiovascular events in people prescribed glucocorticoids with iatrogenic Cushing's syndrome: cohort study. BMJ 2012;345:e4928-e4928.

33. Aviña-Zubieta JA, Abrahamowicz M, Choi HK, et al. Risk of cerebrovascular disease associated with the use of glucocorticoids in patients with incident rheumatoid arthritis: a population-based study. Annals of the Rheumatic Diseases 2011;70:990-995.

34. Rungoe C, Basit S, Ranthe MF, Wohlfahrt J, Langholz E, Jess T. Risk of ischaemic heart disease in patients with inflammatory bowel disease: a nationwide Danish cohort study. Gut 2013;62:689-694.

35. Skov J, Sundström A, Ludvigsson JF, Kämpe O, Bensing S. Sex-Specific Risk of Cardiovascular Disease in Autoimmune Addison Disease—A Population-Based Cohort Study. The Journal of Clinical Endocrinology & Metabolism 2019;104:2031-2040.

36. Ocon AJ, Reed G, Pappas DA, Curtis JR, Kremer JM. Short-term dose and duration-dependent glucocorticoid risk for cardiovascular events in glucocorticoid-naive patients with rheumatoid arthritis. Annals of the Rheumatic Diseases 2021;80:1522-1529.

37. Mapel DW, Nelson LS, Lydick E, Soriano J, Yood MU, Davis KJ. Survival among COPD patients using fluticasone/salmeterol in combination versus other inhaled steroids and bronchodilators alone. COPD 2007;4:127-34.

38. Wei L, MacDonald TM, Walker BR. Taking glucocorticoids by prescription is associated with subsequent cardiovascular disease. Ann Intern Med 2004;141:764-70.

39. Ozen G, Pedro S, Michaud K. The Risk of Cardiovascular Events Associated With Disease-modifying Antirheumatic Drugs in Rheumatoid Arthritis. The Journal of Rheumatology 2021;48:648-655.

40. Persson R, Hagberg KW, Qian Y, Vasilakis-Scaramozza C, Jick S. The risks of major cardiac events among patients with psoriatic arthritis treated with apremilast, biologics, DMARDs or corticosteroids. Rheumatology 2021;60:1926-1931.

41. Greenberg JD, Kremer JM, Curtis JR, et al. Tumour necrosis factor antagonist use and associated risk reduction of cardiovascular events among patients with rheumatoid arthritis. Annals of the Rheumatic Diseases 2011;70:576-582.

42. de Vries F, Pouwels S, Bracke M, et al. Use of inhaled corticosteroids and the risk of non-fatal acute myocardial infarction. J Hypertens 2008;26:124-9.

43. Souverein PC. Use of oral glucocorticoids and risk of cardiovascular and cerebrovascular disease in a population based case-control study. Heart 2004;90:859-865.
