# Supplementary material for: The role of glucocorticoids in increasing cardiovascular risk
Source: Front Cardiovasc Med. 2023 Jul 5;10:1187100. doi: 10.3389/fcvm.2023.1187100 (PMC10354523; doi:10.3389/fcvm.2023.1187100)
Supplement: Supplementary file 4 [file Table2.doc]

Table S2

Quality Assessment of Included Cohort Studies Using the Newcastle-Ottawa Scale

|  | **Selection** | | | | **Comparability** | | **Outcome** | | |  |
| --- | --- | --- | --- | --- | --- | --- | --- | --- | --- | --- |
| **Author** | **Representativeness of**  **Exposed**  **Cohort** | **Selection of Non-Exposed Cohort** | **Ascertainment**  **Of Exposure** | **Demonstration That Outcome**  **of Interest Was Not Present at Start of Study** | **Adjust for age** | **Adjust for other cardiovascular risk factors** | **Assessment of outcome** | **Follow-up**  **length** | **Loss to follow-up rate** | **Total Quality**  **Score** |
| Schultz, 2019 | 1 | 1 | 1 | 0 | 1 | 1 | 1 | 0 | 1 | 7 |
| Yao, 2020 | 1 | 1 | 1 | 0 | 1 | 1 | 1 | 0 | 1 | 7 |
| Innala, 2011 | 1 | 1 | 1 | 0 | 0 | 0 | 1 | 1 | 0 | 5 |
| Cangemi, 2019 | 0 | 1 | 1 | 0 | 1 | 1 | 1 | 0 | 1 | 6 |
| Pujades-Rodriguez, 2020 | 1 | 1 | 1 | 0 | 1 | 1 | 1 | 1 | 1 | 8 |
| Yeh, 2017 | 1 | 1 | 1 | 0 | 1 | 1 | 1 | 0 | 1 | 7 |
| Rincon, 2014 | 1 | 1 | 1 | 1 | 1 | 1 | 1 | 1 | 1 | 9 |
| Kremers, 2007 | 1 | 1 | 1 | 0 | 1 | 0 | 1 | 1 | 1 | 7 |
| Davis, 2007 | 1 | 1 | 1 | 0 | 1 | 1 | 1 | 1 | 1 | 8 |
| Gonzalez-Gay, 2007 | 0 | 1 | 1 | 0 | 1 | 0 | 1 | 1 | 1 | 6 |
| Avina-Zibieta, 2013 | 1 | 1 | 1 | 0 | 1 | 1 | 1 | 1 | 1 | 8 |
| Ellingsen, 2020 | 1 | 1 | 1 | 1 | 0 | 1 | 1 | 0 | 1 | 7 |
| Vollmer, 2009 | 1 | 1 | 1 | 1 | 0 | 0 | 1 | 0 | 1 | 6 |
| Macie, 2006 | 1 | 1 | 1 | 1 | 1 | 1 | 1 | 0 | 1 | 8 |
| Sin, 2003 | 1 | 1 | 1 | 1 | 1 | 1 | 1 | 0 | 1 | 8 |
| Tkacova, 2006 | 0 | 1 | 1 | 1 | 1 | 0 | 1 | 0 | 1 | 6 |
| Sin, 2001 | 1 | 1 | 1 | 1 | 1 | 1 | 1 | 0 | 1 | 8 |
| Fan, 2003 | 1 | 1 | 1 | 1 | 0 | 1 | 1 | 0 | 1 | 7 |
| Shin, 2020 | 1 | 1 | 1 | 0 | 1 | 1 | 1 | 0 | 1 | 7 |
| Thomas， 2021 | 1 | 1 | 1 | 0 | 1 | 1 | 1 | 0 | 1 | 7 |
| Olivarius, 2010 | 0 | 1 | 1 | 1 | 1 | 0 | 1 | 1 | 1 | 7 |
| Cammargo, 2008 | 1 | 1 | 1 | 1 | 1 | 1 | 1 | 1 | 1 | 9 |
| Yeh, 2022 | 1 | 1 | 1 | 1 | 0 | 0 | 1 | 0 | 1 | 6 |
| Fardet, 2012 | 1 | 0 | 1 | 0 | 1 | 1 | 1 | 0 | 1 | 6 |
| Avina-Zibieta, 2011 | 1 | 1 | 1 | 0 | 1 | 1 | 1 | 1 | 1 | 8 |
| Rungoe, 2012 | 1 | 1 | 1 | 1 | 1 | 0 | 1 | 1 | 1 | 8 |
| Skov, 2019 | 1 | 0 | 1 | 0 | 1 | 0 | 1 | 1 | 1 | 7 |
| Ocon , 2021 | 1 | 1 | 1 | 0 | 1 | 1 | 1 | 0 | 1 | 7 |
| Mapel, 2009 | 1 | 1 | 1 | 1 | 1 | 1 | 1 | 0 | 1 | 8 |
| Wei, 2016 | 1 | 1 | 1 | 1 | 1 | 1 | 1 | 0 | 1 | 8 |
| Ozen, 2020 | 1 | 1 | 1 | 0 | 1 | 1 | 1 | 1 | 1 | 8 |
| Persson, 2020 | 1 | 1 | 1 | 1 | 1 | 0 | 1 | 0 | 1 | 7 |
| Greenberg, 2010 | 1 | 1 | 1 | 0 | 1 | 1 | 1 | 0 | 1 | 7 |

The quality of included studies was assessed by the Newcastle Ottawa scale.

**Selection**: 1) Representativeness of exposed cohort: 1, study population truly or somewhat representative of a community/ population based study; 0, study population was sampled from a special population, that is, population from a company, hospital patients, data from the health insurance company or health examination organization, nurses. 2) Selection of non-exposed cohort: 1, drawn from the same community as the exposed cohort. 3) Ascertainment of exposure: 1, Validation of glucocorticoid use with secure medical record; 0, no specific glucocorticoid use validation method. 4) Demonstration that outcome was not present at start of study: 1, exclusion of participants with a history of major adverse cardiovascular events at the beginning of the study.

**Comparability**: 1) 1, whether a study adjusted for age deliberately; 1, whether a study adjusted for other cardiovascular risk factors.

**Outcome**: 1) Assessment of outcome: 1, cardiovascular events were confirmed by medical records or record linkage; 0, self-reported. 2) Was follow-up long enough for outcomes to occur: 1, duration of follow-up >= 5 year; 0, if duration of follow-up < 5 year 3) Loss to follow-up rate: 1, complete follow-up or loss to follow up rate <=20 %; 0, follow-up rate < 80% or no description of those lost.

Quality Assessment of Included Case-Control Studies Using the Newcastle-Ottawa Scale

|  | **Selection** | | | | **Comparability** | | **Outcome** | | |  |
| --- | --- | --- | --- | --- | --- | --- | --- | --- | --- | --- |
| **Author** | **Adequacy of case definition** | **Representativeness of the cases** | **Selection of Controls** | **Definition of Controls** | **Adjust for Age** | **Adjust for Other Cardiovascular Risk Factors** | **Assessment of Outcome** | **Same Method of Ascertainment for Cases and Controls** | **Non-Response Rate** | **Total Quality**  **Score** |
| Blocehliger, 2018 | 1 | 1 | 1 | 0 | 1 | 1 | 1 | 1 | 0 | 7 |
| Suissa, 2006 | 1 | 1 | 1 | 0 | 1 | 1 | 1 | 1 | 0 | 7 |
| Halm, 2006 | 1 | 1 | 0 | 0 | 1 | 0 | 1 | 1 | 0 | 5 |
| Solomon, 2006 | 1 | 1 | 1 | 0 | 1 | 1 | 1 | 1 | 0 | 7 |
| Suissa, 2003 | 1 | 1 | 1 | 0 | 1 | 1 | 1 | 1 | 0 | 7 |
| Huiart, 2005 | 1 | 1 | 1 | 1 | 1 | 1 | 1 | 1 | 0 | 8 |
| Lee, 2008 | 1 | 1 | 1 | 1 | 1 | 0 | 1 | 1 | 0 | 7 |
| Vries, 2008 | 1 | 1 | 1 | 0 | 1 | 0 | 1 | 1 | 0 | 6 |
| Souverein, 2003 | 1 | 1 | 1 | 1 | 1 | 1 | 1 | 1 | 0 | 8 |

The quality of included studies was assessed by the Newcastle Ottawa scale.

**Selection**: 1) Adequacy of case definition: 1, cardiovascular events were confirmed by medical records or record linkage; 0, self-reported. 2) Representativeness of the cases: 1, consecutive or obviously representative series of cases; 0, potential for selection biases or not stated.3) Selection of Controls: 1, community controls; 0, hospital controls or no description.4) Definition of Controls: 1, no history of cardiovascular events; 0, no description of source.

**Comparability**: 1) 1, whether a study adjusted for age deliberately; 1, whether a study adjusted for other cardiovascular risk factors.

**Outcome**: 1) Assessment of outcome: 1, cardiovascular events were confirmed by medical records or record linkage; 0, self-reported. 2) Same method of ascertainment for cases and controls: 1, yes; 0, no. 3) Non-Response rate: 1, same rate for both groups; 0, non respondents described rate different and no designation.

**Quality Assessment of Included Randomized Controlled Studies Using the Modified Jadad Scores**

| **Author** | **Randomization** | **Concealment of**  **Allocation** | **Double Blinding** | **Withdrawals and**  **Dropouts** | **Total** |
| --- | --- | --- | --- | --- | --- |
| Brook, 2017 | 2 | 1 | 2 | 1 | 5 |

**Randomization:** 0, not randomized or inappropriate method of randomization; 1, the study was described as randomized; 2, the method of randomization was described and it was appropriate.

**Concealment of allocation:** 0, not describe the method of allocation concealment; 1, the study was described as using allocation concealment method; 2, the method of allocation concealment was described appropriately.

**Double blinding:** 0, no blind or inappropriate method of blinding; 1, the study was described as double blind; 2, the method of double blinding was described and it was appropriate.

**Withdrawals and dropouts:** 0, not describe the follow-up; 1, a description of withdrawals and dropouts.
