# Supplementary material for: The role of glucocorticoids in increasing cardiovascular risk
Source: Front Cardiovasc Med. 2023 Jul 5;10:1187100. doi: 10.3389/fcvm.2023.1187100 (PMC10354523; doi:10.3389/fcvm.2023.1187100)
Supplement: Supplementary file 5 [file Table3.doc]

**Table S3.** **Prednisolone-equivalent dose conversion factors for glucocorticoids**

| **Glucocorticoid** | **10mg prednisolone-equivalent (in mg)** |
| --- | --- |
| Betamethasone | 1.5 |
| Budenoside | 1.09 |
| Cortisone | 50 |
| Deflazacort | 12 |
| Dexamethasone | 1.5 |
| Hydrocortisone | 40 |
| Methylprednisolone | 8 |
| Prednisone | 10 |
| Triamcinolone | 8 |

Supplemental Table S3
